# Breast Cancer Knowledge and Self‐Examination Practices Among University Female Students in Dinajpur, Bangladesh: A Cross‐Sectional Study

**DOI:** 10.1002/hsr2.71048

**Published:** 2025-07-10

**Authors:** Sabiha Nagnin Hasi, Sumaya Tabassum, Rifah Tamanna Arny, Mst. Dilara Pervin, Md. Golam Hossain, Farhana Hasan, Md. Kaderi Kibria

**Affiliations:** ^1^ Department of Statistics Hajee Mohammad Danesh Science & Technology University Dinajpur Bangladesh; ^2^ Health Research Group, Department of Statistics University of Rajshahi Rajshahi Bangladesh

**Keywords:** awareness, Bangladesh, BC, BSE, risk factors, socioeconomic status

## Abstract

**Background and Aims:**

Breast cancer (BC) is the most common cancer among women worldwide, with increasing incidence rates in developing countries, including Bangladesh. Understanding BC awareness and self‐examination practices among young women is essential for enhancing early detection and prevention efforts. This study aims to assess BC awareness, knowledge of risk factors, and the practice of breast self‐examination (BSE) among female university students in Dinajpur District, Bangladesh.

**Methods:**

A cross‐sectional survey was conducted among 400 undergraduate and postgraduate female students at Hajee Mohammad Danesh Science and Technology University (HSTU) from May 12 to June 20, 2024. Data were collected using a self‐structured questionnaire through face‐to‐face interviews, which collected information on socio‐demographic characteristics, BC awareness, risk factors, and self‐examination practices. Descriptive statistics, percentages, and binary logistic regression were used to analyze the data. SPSS (version 26.0) and R software (version 4.0.2) were employed for statistical analysis.

**Results:**

The study found that 92.4% of participants had heard of BC, with social media being the primary source of information. Although 88.4% recognized the importance of early detection, only 19.1% practiced BC screening, and 2.8% had undergone mammography. While 51.8% reported awareness of BSE, only 4.8% performed it regularly. Significant barriers to BSE included a lack of knowledge (70.7%) and concerns about effectiveness or privacy. Socioeconomic factors were found to significantly influence BC awareness, with rural students and those from lower‐income families exhibiting lower awareness. Additionally, students relying on family income or scholarships had lower awareness compared to those in business.

**Conclusion:**

The study highlights a high level of BC awareness among female university students, but significant gaps remain in self‐examination practices and practical engagement. Socioeconomic status and income sources are key factors influencing BC awareness. Targeted educational interventions are needed to address these gaps and improve early detection and preventive practices among female university students.

**Abbreviations**
BC: breast cancer
BHGI: Breast Health Global Initiative
BSE: breast self‐examination
CBE: clinical breast examination
HELLP: hemolysis, elevated liver enzymes, and low platelets
HSTU: Hajee Mohammad Danesh Science and Technology university
OR: odds ratio
PPS: probability proportion to size

## 1 Introduction

Breast cancer (BC) is the most prevalent cancer among women globally, with a particularly high incidence in developing countries like Bangladesh. This malignancy originates in the breast cells and contributes significantly to the rising mortality rates among women worldwide [1]. In 2020, BC affected approximately 2.3 million women, accounting for 11.7% of all newly diagnosed cancers globally [2, 3, 4]. The annual impact of BC on women is estimated at 2.1 million, with projections indicating a rise to an additional 3.2 million women affected by 2050 [5]. Alarmingly, projections suggest that this number could rise to 3.2 million women by 2050, indicating an urgent need for effective prevention and early detection strategies [6].

South Asian countries, including Bangladesh, are facing a burgeoning BC crisis [7]. In 2023, BC accounted for 45% of all cancer cases among Asian women, reflecting a notable increase from previous years [8]. Bangladesh, a densely populated and developing nation, is particularly vulnerable due to a combination of rapid population growth and a lack of widespread awareness about breast cancer. Over the past 5 years, the incidence of breast cancer in Bangladesh has escalated, with breast cancer now contributing to 32.8% of female cancer cases and resulting in 69% of female cancer‐related deaths [9]. In 2023 alone, 150,781 new cases were reported among women in the country [10].

The burden of breast cancer in Bangladesh is further exacerbated by the late‐stage diagnosis, with 90% of cases being detected at stage three or four [11]. This troubling trend is largely attributed to the lack of awareness about breast cancer, limited access to health services, and insufficient knowledge of BSE as a critical early detection method [12]. Modifiable risk factors such as early menarche, hormone replacement therapy, late menopause, alcohol consumption, obesity, and physical inactivity also play a significant role in the increasing incidence of BC in the country [13, 14]. Furthermore, BC is most commonly identified by two symptoms: a lump in the breast and bloody discharge from the nipple [15].

Breast self‐examination (BSE) is a cost‐effective, noninvasive method that empowers women to detect early changes in breast tissue, which may lead to the early diagnosis of breast cancer. Studies conducted in Jordan have highlighted the critical role BSE plays in enhancing awareness and encouraging proactive health behavior, especially among women studying or working in medical fields [16, 17]. However, despite its recognized importance, the practice of BSE remains suboptimal, even among medically knowledgeable populations, due to factors such as limited understanding of proper techniques, misconceptions about effectiveness, and socio‐cultural barriers. A study in North Jordan also emphasized the gaps in knowledge regarding breast cancer causes and the advantages of medical imaging, which further reflects broader limitations in breast health literacy [17, 18]. These findings underscore the need for structured education on BSE, integrated into both academic and public health initiatives, particularly in settings where access to diagnostic imaging and mammography may be limited or stigmatized. Thus, promoting BSE can serve as an essential strategy in low‐resource settings like Bangladesh to encourage early detection and improve breast cancer outcomes.

Despite the high incidence rates, there is a noticeable gap in knowledge and education regarding BC risk factors and BSE practices, particularly among younger women and those in reproductive age groups. Although BC is more prevalent among women over 50, the incidence among younger women under 50 is rising, possibly due to a reluctance to seek medical help and a lack of awareness among older women. Early detection through methods like BSE, mammography, and clinical breast examinations is crucial in managing the disease more effectively [19]. According to the Breast Health Global Initiative (BHGI), widespread knowledge and practice of BSE can lead to earlier detection and better disease outcomes, especially in resource‐limited settings like Bangladesh [20].

In Bangladesh, few studies have focused on the awareness and practice of BSE among young, educated women, particularly female university students, who represent a critical demographic for BC prevention efforts. Previous research conducted at a university in Dhaka indicated a low prevalence of BSE among students, underscoring the need for further investigation at the district level [21]. Therefore, this study aims to assess BC awareness, risk factors, and BSE practices among female university students in the Dinajpur district of Bangladesh, with the goal of identifying gaps in knowledge and promoting early detection practices within this population.

## 2 Methods

### 2.1 Study Design and Setting

A cross‐sectional study was conducted among all undergraduate and postgraduate female students of HSTU, located in the Dinajpur district, a rural town in the northern part of Bangladesh. The survey took place from May 12, 2024, to June 20, 2024. HSTU comprises nine faculties and 45 departments, with nearly 11,000 students enrolled, a significant portion of whom are female. The inclusion criteria for the study were all female HSTU students from any academic year who were present during the data collection period and willing to participate. The exclusion criteria included male students and any female students less than 18 years of age. Only participants who were willing to participate in the study were included.

### 2.2 Participants

**Figure 1:**
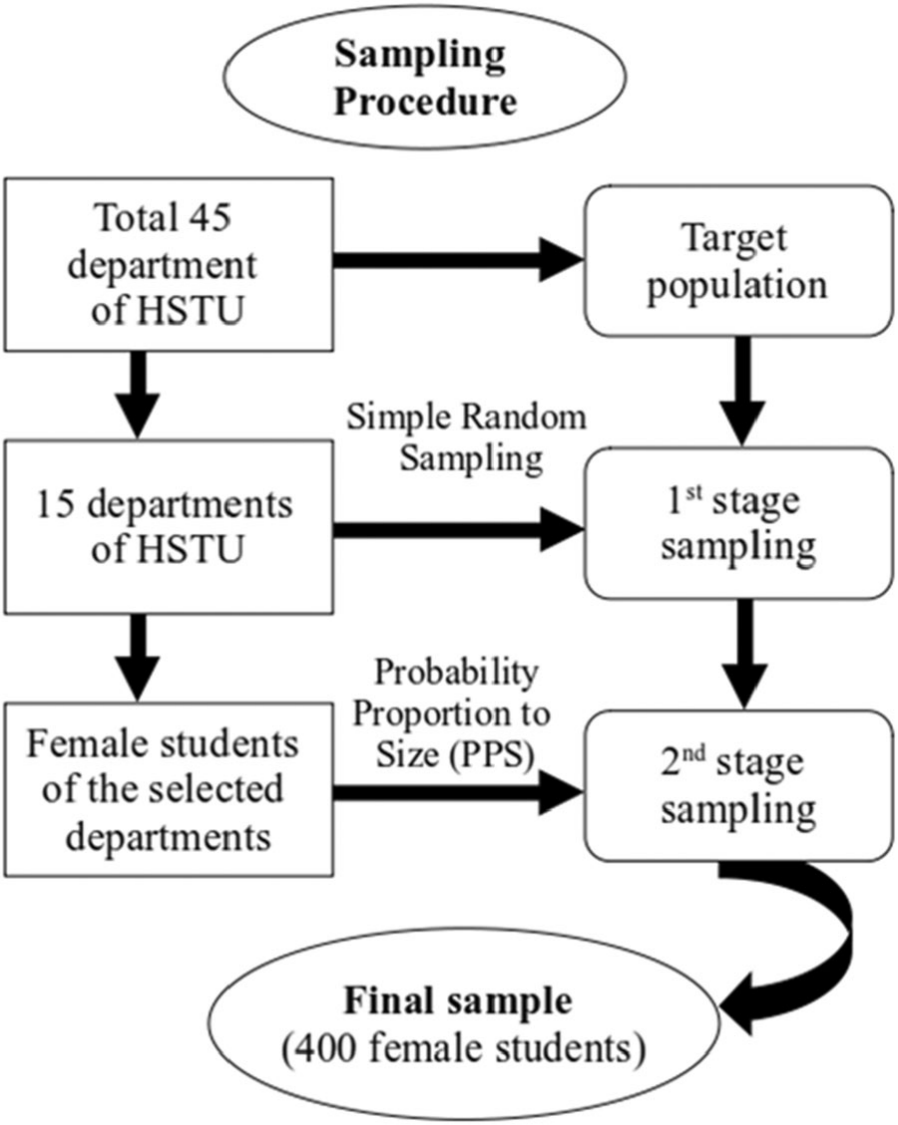
Sampling design for the study.

A two‐stage sampling approach was employed to select participants (see Figure 1). In the first stage, 15 departments were randomly selected from the 45 departments at HSTU using simple random sampling. In the second stage, approximately 400 female students were selected from these departments using probability proportional to size (PPS). The sample size was increased to 400, exceeding the minimum required sample of 384, to enhance the precision of the analysis. Eligibility criteria included all undergraduate and postgraduate female students from the selected departments who were present during the data collection period and voluntarily agreed to participate. Male students and female students under 18 years of age were excluded from the study. To focus on BC awareness, students who had not heard of BC were excluded from further analysis, as the subsequent questions required a basic level of awareness to yield relevant responses.

### 2.3 Sample Size Determination

The required sample size for this study was obtained by using Cochran's formula for a single proportion: $n=Z^{2}p\left(1-p\right)/e^{2};$ where *n* is the required sample size, *Z* indicates the z score corresponding to a 95% confidence interval (CI), *p* is the estimated proportion of the attribute in the population, which is 0.50, and *e* is the margin of error, which is considered 5% in this study [22, 23]. Based on these parameters, the calculated sample size was 384. To ensure a sufficient sample and account for potential non‐responses, we surveyed 400 students, all of whom participated.

### 2.4 Variables and Measurement

A self‐structured questionnaire was developed based on a comprehensive literature review. Before the main data collection, a pilot study was conducted with 60 participants who shared similar demographic characteristics with the target population but were not included in the final study sample. The pilot study aimed to evaluate the clarity, reliability, and acceptability of the questionnaire items. Necessary modifications were made based on the feedback received. Furthermore, the questionnaire was reviewed by subject matter experts in statistics and public health to establish content validity. This questionnaire was used to collect data from participants through face‐to‐face interviews. It was designed to gather information on (i) sociodemographic characteristics, (ii) sources of information, (iii) awareness of BC, (iv) signs and symptoms of BC, (v) risk factors, and (vi) knowledge and practices related to BSE. Printed copies of the questionnaire were provided to respondents, who were given time to complete them. Before distributing the questionnaires, the objectives of the study were explained to the participants. Only female university students at HSTU who consented to participate were included in the study. Written consent was obtained from each participant.

### 2.5 Statistical Analysis

Data from completed questionnaires were analyzed using SPSS (version 26.0), and graphical representations were generated using R software (version 4.0.2). Descriptive statistics were employed to summarize the data set. The chi‐square test (two‐tailed) was conducted to examine associations between demographic variables and knowledge of breast cancer awareness, symptoms, and risk factors. Binary logistic regression was performed to identify significant predictors of knowledge levels. Before the regression analysis, multicollinearity among independent variables was assessed using the variance inflation factor (VIF), with all values falling within acceptable limits. The strength of associations was presented as adjusted odds ratios (aORs) with corresponding 95% confidence intervals (CIs), adjusting for potential confounding variables to provide more accurate estimates.

### 2.6 Ethics Approval and Consent to Participate

This study was conducted in accordance with the principles outlined in the Declaration of Helsinki and was approved by the Institutional Animal, Medical Ethics, Biosafety, and Biosecurity Committee (IAMEBBC) of the Institute of Biological Sciences, University of Rajshahi, Rajshahi‐6205, Bangladesh with ethical approval number: 211/320/(69) | AMEBBC/|BSc. Written informed consent was obtained from all participants before to data collecting.

## 3 Results

### 3.1 Descriptive Statistics

#### 3.1.1 Demographic Profiles

The study sample predominantly comprised participants aged 20–24 years (78.8%), with the majority coming from rural areas (63.1%). Most of them identified as Muslim (88.2%) and were unmarried (90.2%), with a significant portion residing in university halls (62.1%). In terms of socioeconomic status, the majority of families had an income between 20,000 and 50,000 (60.2%), with family support serving as the primary income source for 87.9% of students. Additionally, most participants experiences menarche between the ages of 12–15 (81.4%), and only a few reported a family history of BC (7.8%) (see Table 1).

**Table 1:** Demographic characteristics of the participants (*n* = 400).

Variable	Frequency	Percentage	Variable	Frequency	Percentage
Age (years)			Family income		
Less than 20	26	6.5	Less than 20000	95	25.5
20–24	313	78.8	20,000–50,000	224	60.2
More than 24	58	14.6	More than 50000	53	14.2
Birthplace			Age of menarche		
Rural	251	63.1	12–15	324	81.4
Urban	142	35.7	Above 15	64	16.1
Religion			Family history of BC		
Muslim	351	88.2	Yes	31	7.8
Hindus	46	11.6	No	352	84.4
Marital status			Don't know	8	2.0
Married	39	9.8	Your income source		
Unmarried	359	90.2	Family	350	87.9
Current residential status			Scholarship	6	1.5
Hall	247	62.1	Tuition	32	8.0
Mess	104	26.1	Business	6	1.5
Others	45	11.3			

**Figure 2:**
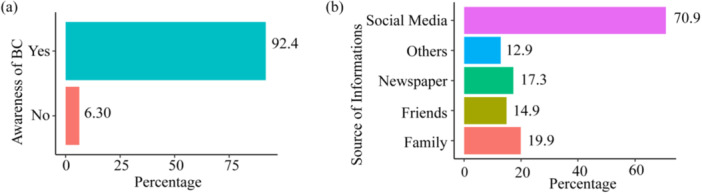
Bar plot. (a) Awareness of breast cancer among students, (b) Source of information on breast cancer.

Nearly all students (92.4%) had heard about breast cancer, with social media (70.9%) being the primary source, followed by family (19.9%), newspapers (17.3%), and friends (14.9%) (see Figure 2). Notably, the 7.6% of the students who had not heard about BC were excluded from further analysis.

#### 3.1.2 Knowledge of Awareness and Symptoms

**Figure 3:**
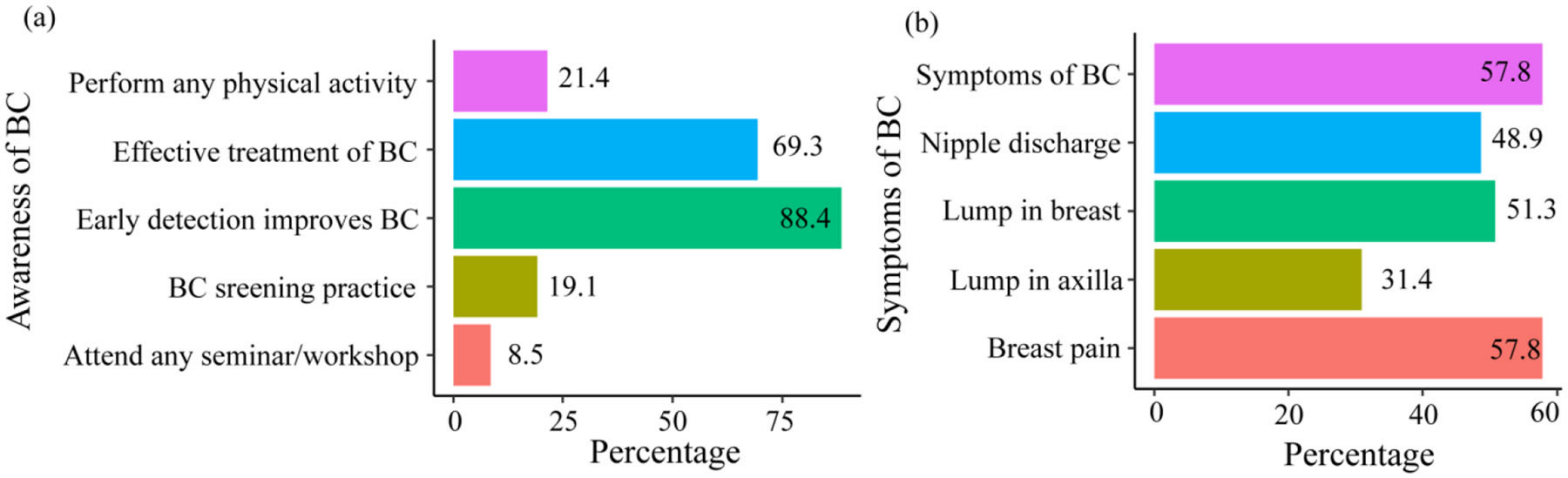
Knowledge of breast cancer awareness and symptoms among university students: (a) General awareness, (b) symptoms recognition.

The study revealed that although a significant majority of participants recognized the importance of early detection in improving breast cancer treatment (88.4%) and its overall benefits (94.2%), there were notable gaps in practical engagement. Only 19.1% of participants practiced BC screening, and just 2.8% had undergone mammography. Awareness of BSE was nearly evenly split, with 49.0% of participants having heard of it. Moreover, while 69.3% believed in the effectiveness of BC treatment, very few had attended seminars or workshops (8.5%), were aware of clinical breast examinations (15.1%), or engaged in physical activity (21.4%) (see Figure 3a). The results also show varying levels of awareness regarding common BC symptoms. A substantial proportion of participants recognized symptoms such as a lump in the breast (50.8%) and breast pain (57.8%), but fewer identified a lump in the axilla (30.9%) and nipple discharge (48.7%). These findings suggest that while awareness of some symptoms is relatively high, there is a significant gap in recognizing less common symptoms, underscoring the need for more comprehensive educational efforts on all potential breast cancer symptoms (see Figure 3b).

#### 3.1.3 Knowledge of Risk Factors and BSE

**Figure 4:**
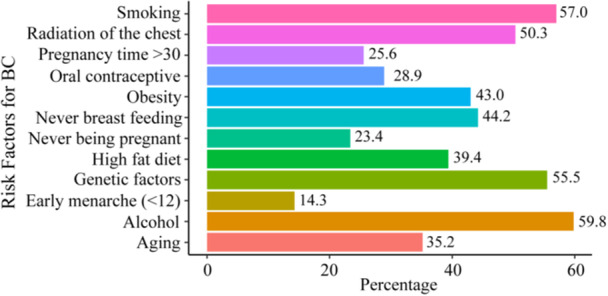
Knowledge of breast cancer risk factors among university students who identified them.

Varying levels of awareness among students regarding different risk factors for BC were shown in Figure 4. The most recognized risk factors include alcohol consumption (59.8%), smoking (57.0%), and genetic factors (55.5%). Radiation to the chest was identified by 50.3% of the students, and obesity was recognized by 43.0%. High‐fat diet and lack of breastfeeding were also noted by 39.4% and 44.2% of students, respectively. However, awareness of some factors was relatively lower; only 28.9% identified oral contraceptive use, 25.6% were aware of the risk associated with pregnancy after age 30, 23.4% recognized the risk of never being pregnant, and just 14.3% were aware of the risk associated with early menarche (before age 12).

Regarding BSE, 51.8% of participants reported being aware of BSE, while 36.7% were not familiar with it, and 11.1% were unsure (see Supporting Information S1: Figure S1). Among those aware, 11.1% expressed concern about the timing of BSE, 61.1% were not worried about it, and 26.9% were uncertain. A significant 84.4% of participants had never performed BSE, 4.8% performed it regularly, and 9.3% did so irregularly. Various reasons were cited for not performing BSE: 70.7% did not know how to perform it, 9.5% lacked time, 7.6% were uninterested, and 4.5% feared receiving a positive result. Additionally, 9.3% were unsure of BSE's effectiveness in detecting issues, and 3.3% cited a lack of privacy as a concern.

### 3.2 Socio‐Demographic Determinants of BC Knowledge and BSE

The study examines the relationships between key demographic variables and the knowledge of BC awareness, symptoms, and risk factors among female students aware of BC (see Supporting Information S1: Table S1‐S3). The results demonstrate a high awareness of the importance of early detection across all demographics, with 88.7%–97.3% of students recognizing its significance. However, participation in BC seminars was low, with less than 12% attendance in most groups. Urban students and those with higher family incomes exhibited greater engagement in breast screening and physical activity, which are crucial for prevention. In terms of symptom recognition, the most commonly identified symptoms were lumps in the breast and breast pain, with urban residents and wealthier students showing higher awareness. Conversely, awareness of less obvious symptoms, such as lumps in the axilla and nipple discharge, was generally lower, particularly among rural students. Personal experiences, such as being married or having a family history of BC, correlated with a higher understanding of multiple symptoms.

The chi‐square test results further highlight significant associations between certain demographic variables and BC knowledge (see Table 2). Urban students demonstrated significantly higher awareness (*p* = 0.015) compared to rural students, and those from higher‐income families ( > 500,000 Tk) had greater awareness (*p* = 0.027) than students from lower‐income groups. Students relying on scholarships also exhibited significantly higher awareness (*p* = 0.020) and a better understanding of risk factors (*p* = 0.006) compared to those supported by family income or tuition. However, no significant associations were found between age, religion, marital status, or age of menarche and BC awareness, symptoms, or risk factors. Although urban students and those with higher family incomes tended to practice BSE more frequently, these differences were not statistically significant. These findings underscore the influence of Socioeconomic factors, particularly place of birth and income, on BC knowledge, while other demographic variables appear to have less impact, highlighting areas where targeted education efforts could be beneficial.

**Table 2:** The relationships between demographic variables and the knowledge of BC awareness, symptoms, and risk factors among female students who are aware of BC (*n* = 367).

Variables	Awareness			Symptoms			Risk factors			Perform BSE		
	No	Yes	*p* value	No	Yes	*p* value	No	Yes	*p* value	No	Yes	*p* value
Age in years												
< 20	73.9	26.1	0.930	39.1	60.9	0.608	56.5	43.5	0.837	100	0.0	0.249
20‐24	71.7	28.3		39.3	60.7		57.2	42.8		94.1	5.9	
> 24	69.8	30.2		41.5	58.5		52.8	47.2		98.1	1.9	
Birth place												
Rural	76.1	23.9	0.015	42.2	57.8	0.333	56.5	43.5	0.953	96.5	3.5	0.101
Urban	64.2	35.8		35.0	65.0		56.2	43.8		92.7	7.3	
Residence												
Hall	72.4	27.6	0.847	44.4	55.6	0.040	57.8	42.2	0.254	94.7	5.3	0.563
Mess	71.4	28.6		33.7	66.3		50.0	50.0		96.9	3.1	
Others	68.2	31.8		27.3	72.7		63.6	36.4		93.2	6.8	
Religion												
Muslim	72.0	28.3	0.659	39.4	60.6	0.943	56.5	43.5	0.903	94.7	5.3	0.374
Hindus	68.9	31.1		40.0	60.0		55.6	44.4		97.8	2.2	
Marital status												
Unmarried	72.4	27.6	0.333	40.9	59.1	0.101	57.6	42.4	0.176	95.5	4.5	0.341
married	64.9	35.1		27.0	73.0		45.9	54.1		91.9	8.1	
Age of menarche												
12–15 years	71.3	28.7	0.737	38.2	61.8	0.217	55.7	44.3	0.528	95.2	4.8	0.783
> 15 years	73.6	26.4		47.2	52.8		60.4	39.6		94.3	5.7	
Family history of BC												
Yes	71.5	28.5	0.800	39.9	60.1	0.598	56.5	43.5	0.949	95.5	4.5	0.267
No	73.5	26.5		35.3	64.7		55.9	44.1		91.2	8.8	
Family income (tk)												
< 20,000	77.4	22.6	0.027	38.1	61.9	0.294	56.0	44.0	0.175	97.6	2.4	0.100
20,000‐50,000	73.7	26.3		42.6	57.4		59.8	40.2		95.7	4.3	
> 500,000	59.5	40.5		32.4	67.6		47.3	52.7		90.5	9.5	
Income source												
Family	72.4	27.1	0.020	40.5	59.5	0.245	56.7	43.3	0.714	95.0	5.0	0.006
Scholarship	60.0	40.0		10.0	90.0		40.0	60.0		100	0.0	
Tuition	73.3	26.7		36.7	63.3		56.7	43.3		100	0.0	
Business	16.7	83.3		50.0	50.0		66.7	33.3		66.7	33.3	

### 3.3 Predictors of BC Awareness

**Figure 5:**
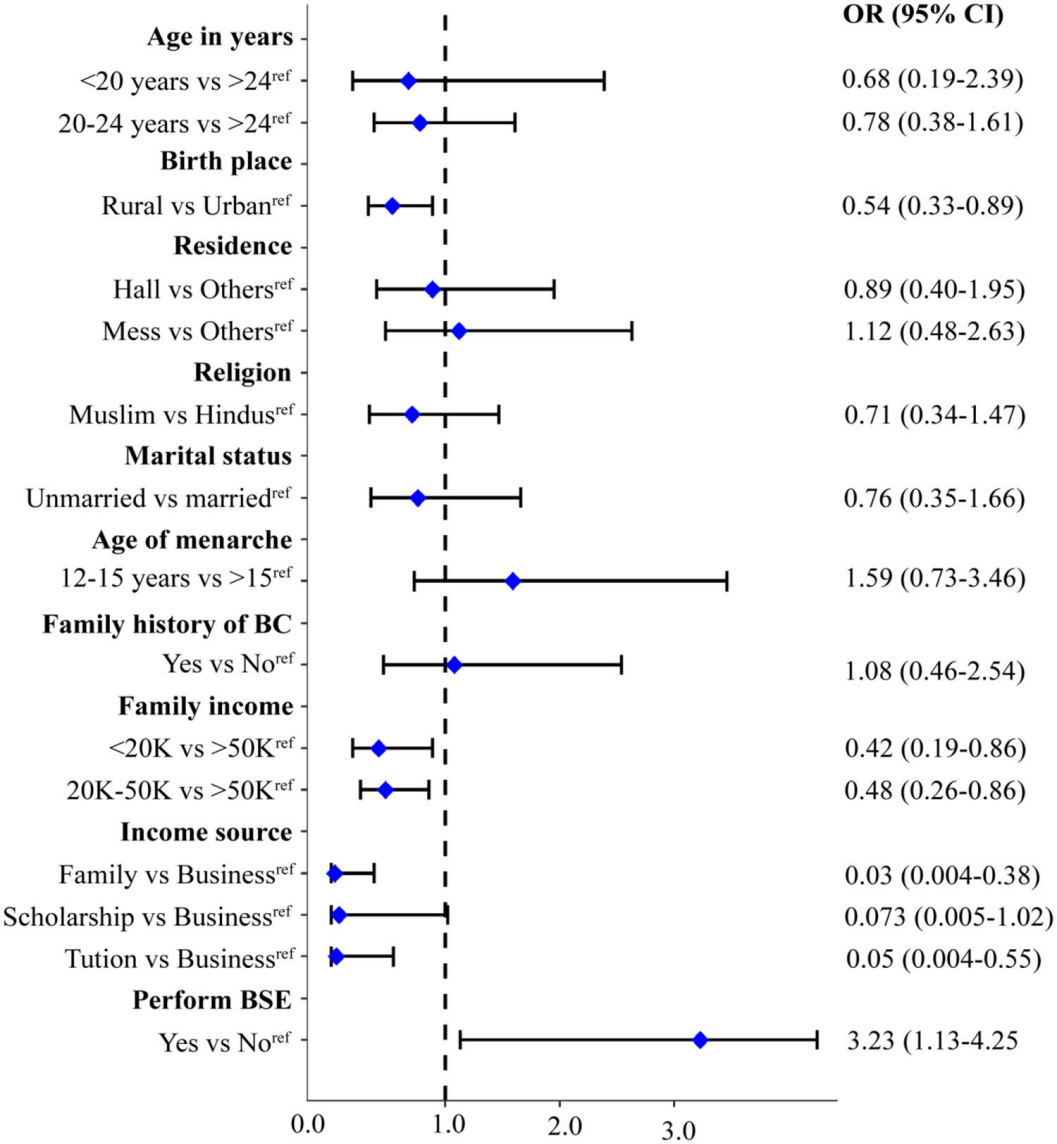
The forest plot illustrates the predictor of BC awareness among the university female students.

The binary logistic regression analysis revealed significant predictors of BC awareness among female students (see Figure 5). Students from rural areas were notably less aware of BC compared to their urban counterparts (aOR = 0.54, 95% CI: 0.33–0.89). Socioeconomic factors also played a crucial role, with students from lower‐income families ( < 20,000 Tk and 20,000–50,000 Tk) showing significantly lower odds of awareness (aOR = 0.42, 95% CI: 0.19–0.89 and aOR = 0.48, 95% CI: 0.26–0.86, respectively) compared to those from higher‐income families ( > 500,000 Tk). Additionally, students relying on family income, scholarships, or tuition had substantially lower odds of BC awareness (aOR = 0.039, 95% CI: 0.004–0.38; aOR = 0.073, 95% CI: 0.005–1.02; and aOR = 0.05, 95% CI: 0.004–0.55, respectively) compared to those engaged in business. Furthermore, not performing BSE was associated with significantly lower odds of BC awareness (aOR = 0.309, 95% CI: 0.108–0.883).

In contrast, variables such as age, marital status, religion, age of menarche, and family history of BC did not show significant associations with BC awareness. These findings highlight the critical influence of socioeconomic status, income source, and personal health practices on BC awareness among female students.

## 4 Discussion

Breast cancer is a widespread malignant disease in the female population and is considered the leading cause of female mortality worldwide. Awareness of BC and the adoption of preventive practices, such as regular self‐examinations and screenings, are critical for early detection and improving treatment outcomes [24]. However, this study reveals significant gaps in knowledge and engagement with BC prevention among female university students in the Dinajpur District of Bangladesh.

The study found that 92.4% of students had heard about BC, a level of awareness comparable to findings from studies conducted in the UAE and other parts of Bangladesh [4, 25]. The participants in our study identified social media as the main source of information about BC, which is consistent with the findings of other studies [21, 26, 27, 28, 29, 30, 31]. This underscores the potential of digital platforms in disseminating crucial health information, yet also highlights the need to ensure that the content is accurate, comprehensive, and reaches underserved populations. While social media is a useful platform, relying solely on it may not address the information needs of all students, particularly those from rural areas or low socioeconomic backgrounds. Therefore, university‐based health education campaigns led by professionals or peer educators may complement digital platforms with evidence‐based and culturally sensitive content.

Despite the high awareness of BC as a disease, there were concerning gaps in the knowledge of specific symptoms and risk factors. For instance, while breast pain was commonly recognized (57.8%), awareness of other symptoms such as lumps in the axilla (30.9%) and nipple discharge (48.7%) was markedly lower. This disparity suggests that while students may be aware of BC in general, their understanding of the full spectrum of symptoms is limited. These findings are consistent with studies conducted in Pakistan and Egypt, where similar trends were observed [25, 26, 32, 33]. This lower recognition of certain symptoms, especially among students from rural or lower socioeconomic backgrounds, highlights the need for targeted educational interventions that address these specific gaps. Incorporating interactive learning strategies such as demonstrations, audiovisual tools and real‐life testimonials in university curricular could help bridge these knowledge gaps and enhance symptom recognition.

Regarding the risk factors, the study revealed that a significant portion of students lacked awareness of key factors such as the use of oral contraceptives, pregnancy‐related risks, and early menarche [34, 35, 36, 37]. While more than half of the participants recognized the risks associated with smoking, genetic factors, and radiation exposure, less than 30% were aware of the risks related to oral contraceptive use and pregnancy after age 30 [26]. These findings align with prior research indicating a general lack of knowledge about less apparent risk factors among young women in similar settings [38, 39]. The stark contrast in awareness between common and less recognized risk factors suggests that current educational efforts may not be sufficiently comprehensive, particularly in addressing the nuances of BC risk. Future research could benefit from qualitative approaches to explore why these risk factors remain poorly understood and how educational content can be tailored to enhance comprehension of these issues.

Moreover, the study highlights a low level of engagement in preventive practices such as BSE and clinical breast examinations (CBE). Although nearly half of the students were aware of BSE, only 19.1% practiced it, and a mere 4.8% performed it regularly. This level of engagement is consistent with findings from studies in Bangladesh, Egypt, and other regions where similar rates of BSE practice were reported [21, 40]. The prevalence of BSE among female students is notably low in several countries, including Iraq (19.7%), Turkey (20.3%), Egypt (6.1%), Cameroon (38.5%), and Yemen (17.4%) [19, 26, 41, 42, 43]. The barriers to performing BSE identified in this study, including lack of knowledge, time constraints, and fear reflect those found in similar studies across different cultural contexts [25, 26, 44]. These barriers suggest that simply raising awareness is not enough; there must be a concerted effort to provide practical education on how to perform BSE, alongside reassurance about its efficacy and importance [45]. The integration of health behavior models such as the Health Belief Model (HBM) could be instrumental in designing interventions that explore and address students perceived susceptibility, perceived barriers and motivational triggers to perform BSE.

Socioeconomic factors, particularly residence and family income, were found to be significant determinants of both awareness and engagement in preventive practices [46]. Students from rural areas and lower‐income families exhibited notably lower levels of awareness and were less likely to engage in BSE. This finding is consistent with research highlighting the influence of socioeconomic status on health behaviors, suggesting that interventions should be tailored to address these disparities [47, 48, 49]. While socioeconomic factors play a significant role in shaping breast cancer awareness and screening practices, specific interventions can be designed to target these disparities. Students from lower‐income families and rural areas often face challenges in accessing mammograms and other health check‐up services due to financial and logistical barriers [50, 51]. Therefore, implementing initiatives such as providing scholarships for mammograms, organizing mobile screening programs, and offering free health check‐ups could significantly improve early detection rates among these vulnerable populations. Additionally, community‐based interventions such as peer‐led educational sessions, tailored to the needs of rural or socio‐economically disadvantaged students, could further enhance awareness [52]. These targeted interventions, along with equitable resource distribution, would ensure that individuals from all backgrounds have equal access to breast cancer prevention and screening services. Further disaggregation of socioeconomic indicators such as parental education or occupation could also help to develop more nuanced strategies for engagement and resource allocation.

Despite a moderate to high level of awareness regarding BC and its early detection methods among participants, practical engagement in screening practices particularly BSE and mammography remains limited. This highlights the need for practical, hands‐on educational interventions, such as structured workshops focused on proper BSE techniques [53, 54]. Collaborations with healthcare providers could further enhance access to screening services, especially for students from socio‐economically disadvantaged backgrounds [55]. Integrating health behavior models, such as the Health Belief Model (HBM), may provide a valuable theoretical framework to explore perceived susceptibility, perceived benefits, and barriers to screening. Applying such models can help design targeted educational strategies that more effectively transform awareness into proactive health behavior. We recommend that university health programs incorporate tailored educational initiatives on breast cancer awareness, BSE, and the significance of regular screening, particularly aimed at addressing the specific needs of students from rural and low‐income communities to ensure broader impact and accessibility. Future research should also explore student's personal beliefs, attitudes and motivators behind engaging or not engaging in BSE to develop interventions that are both effective and culturally appropriate.

## 5 Limitations

This study has several limitations. Firstly, it focuses exclusively on a single rural university, which may limit the generalizability of the findings to other populations. Additionally, the exclusion of students who had not heard of BC may introduce selection bias, restricting the results to those already familiar with the disease. The reliance of self‐reported knowledge and perceptions could also lead to recall bias or social desirability bias, potentially affecting the accuracy of the data. Furthermore, the absence of comparative data from urban universities or other regions limits the ability to fully understand the broader context of BC awareness. Although probability sampling techniques, including SRS and PPS, were used to minimize selection bias, non‐sampling biases such as nonresponse or coverage errors may still affect the results generalizability. Moreover, the study didn't explore in depth the underlying psychological, cultural and emotional barriers that may prevent students from engaging in BSE or seeking clinical screening services. Incorporating qualitative methods such as in‐depth interviews or focus groups in future research would allow more holistic understanding of these barriers. Additionally, while socioeconomic status was found to be a determinant of awareness, more granular analysis of its components (e.g., parental education, household income, access to healthcare) would strengthen the interpretation of its influence. Further research should consider longitudinal studies to track changes in awareness and practices over time, as well as evaluate the effectiveness of targeted educational interventions.

## 6 Conclusion

This study provides valuable insights into the awareness, knowledge, and practices related to BC among female university students in the Dinajpur District of Bangladesh. Although a moderate level of awareness regarding BC symptoms and risk factors was observed, there remains a substantial gap in the actual practice of BSE and participation in screening programs. Socio‐demographic factors such as place of residence, family income, and educational background were found to significantly influence both awareness and engagement in preventive behaviors. Students from rural areas and lower‐income families exhibited lower knowledge levels and were less likely to practice BSE. These findings underscore the urgent need for comprehensive and culturally sensitive educational interventions to enhance BC awareness and promote early detection, particularly among vulnerable and underserved populations. Strengthening BC education within university settings, coupled with broader community outreach, can play a vital role in reducing the future burden of breast cancer in Bangladesh. Future efforts should focus on practical BSE training, expanding access to screening services through healthcare collaborations, and employing theory‐based approaches such as the Health Belief Model to better understand and address behavioral barriers to breast cancer prevention.

## Author Contributions

**Sabiha Nagnin Hasi:** conceptualization, methodology, formal analysis, writing – original draft, data curation, software. **Sumaya Tabassum:** methodology, software, data curation, formal analysis, conceptualization, writing – original draft. **Rifah Tamanna Arny:** software, methodology, data curation, formal analysis, visualization, writing – original draft. **Mst Dilara Pervin:** writing – review and editing. **Md Golam Hossain:** writing – review and editing, validation, investigation, supervision. **Farhana Hasan:** writing – review and editing, validation. **Md Kaderi Kibria:** conceptualization, methodology, software, investigation, validation, formal analysis, supervision, project administration, writing – review and editing, resources.

## Conflicts of Interest

The authors declare that they have no conflicts of interest.

## Transparency Statement

The lead author Md. Kaderi Kibria affirms that this manuscript is an honest, accurate, and transparent account of the study being reported; that no important aspects of the study have been omitted; and that any discrepancies from the study as planned (and, if relevant, registered) have been explained.

## Supporting information

Supplimentary file.

## Data Availability

The data that support the findings of this study are available on request from the corresponding author. The data are not publicly available due to privacy or ethical restrictions. The datasets used and/or analyzed during this study are available from the corresponding author upon reasonable request.
