# Supplementary material for: Breast Cancer Knowledge and Self‐Examination Practices Among University Female Students in Dinajpur, Bangladesh: A Cross‐Sectional Study
Source: Health Sci Rep. 2025 Jul 10;8(7):e71048. doi: 10.1002/hsr2.71048 (PMC12242694; doi:10.1002/hsr2.71048)
Supplement: Supplementary file 1 — Supplimentary file. [file HSR2-8-e71048-s001.docx]

Breast Cancer Knowledge, Risk Factors and Self-Examination Among Female University Students in Dinapur, Bangladesh


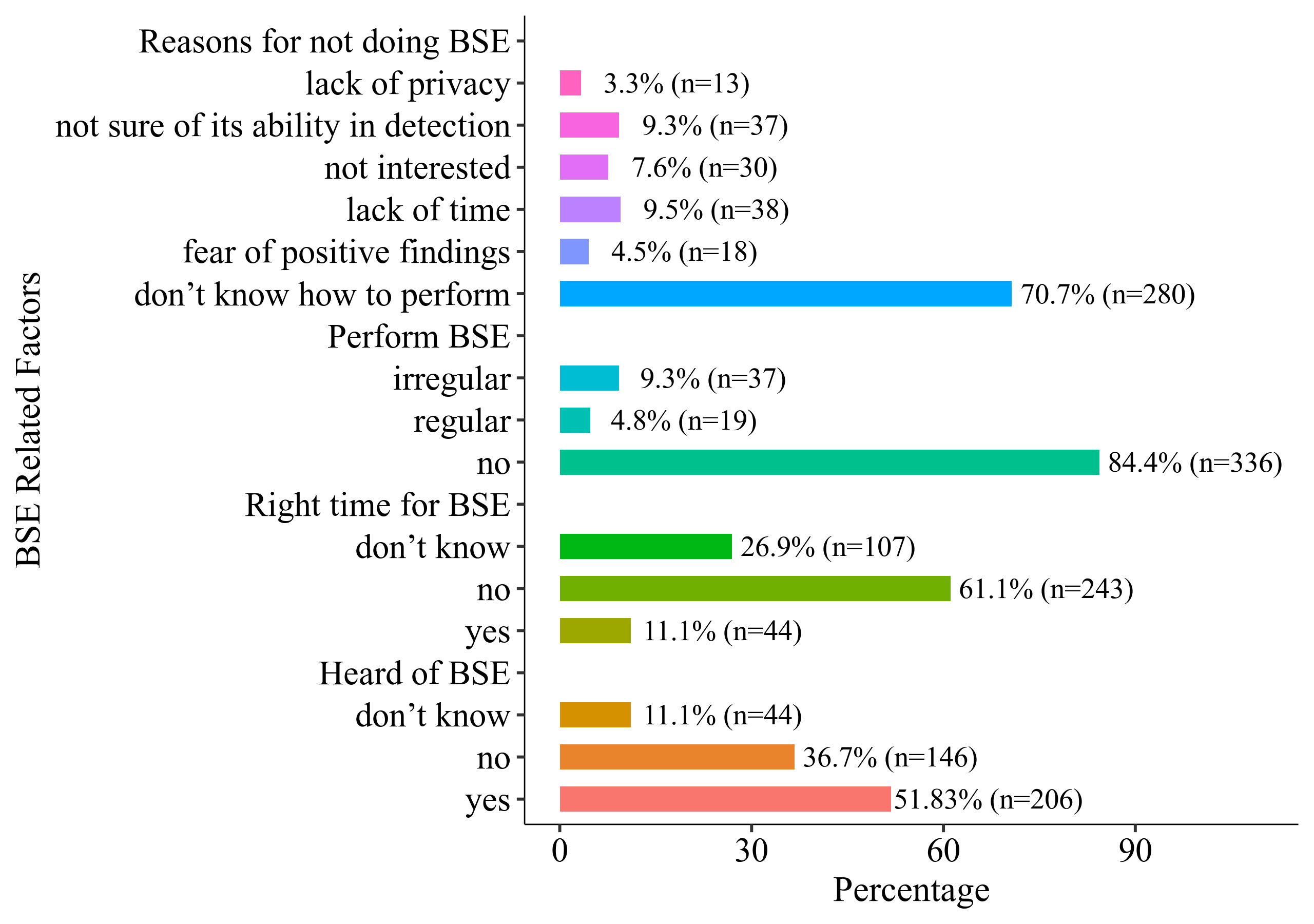
Figure S1: Knowledge and practice of breast self-examination (BSE)

Table S1: Knowledge of awareness of BC among the female students who are aware of BC (N=367)

| **Variables** | **Early detection improve treatment** | **Effective treatment of BC** | **Attending any seminar on BC** | **Breast screening**  **practice** | **Perform physical activities** | **N** |
| --- | --- | --- | --- | --- | --- | --- |
| **Age in years** | | | | | | |
| Less than 20 | 91.3 | 73.9 | 0.0 | 8.7 | 17.4 | 23 |
| 20-24 | 89.7 | 71.7 | 9.0 | 22.8 | 21.4 | 290 |
| More than 24 | 92.5 | 69.8 | 11.3 | 15.1 | 26.4 | 53 |
| **Birth place** | | | | | | |
| Rural | 89.6 | 70.9 | 8.7 | 17.0 | 13.9 | 230 |
| Urban | 91.2 | 73.0 | 8.8 | 27.0 | 35.0 | 137 |
| **Residence** | | | | | | |
| Hall | 89.8 | 70.7 | 8.9 | 20.9 | 19.1 | 225 |
| Mess | 91.8 | 71.4 | 6.1 | 20.4 | 27.6 | 98 |
| Others | 88.6 | 77.3 | 13.6 | 20.5 | 22.7 | 44 |
| **Religion** | | | | | | |
| Muslim | 90.4 | 72.0 | 8.7 | 79.5 | 21.1 | 322 |
| Hindus | 88.9 | 68.9 | 8.9 | 77.8 | 26.7 | 45 |
| **Marital status** | | | | | | |
| Unmarried | 89.4 | 70.6 | 8.5 | 19.7 | 21.5 | 330 |
| married | 97.3 | 81.1 | 10.8 | 29.7 | 24.3 | 37 |
| **Age of menarche** | | | | | | |
| 12-15 years | 90.4 | 71.7 | 7.6 | 20.4 | 21.7 | 314 |
| >15 years | 88.7 | 71.7 | 15.1 | 22.6 | 22.6 | 53 |
| **Family history of BC** | | | | | | |
| Yes | 91.2 | 79.4 | 8.8 | 17.6 | 20.6 | 34 |
| No | 89.8 | 70.2 | 8.3 | 20.6 | 21.5 | 333 |
| **Family income (tk)** | | | | | | |
| <20000 | 88.1 | 66.7 | 8.3 | 16.7 | 17.9 | 84 |
| 20000-50000 | 90.0 | 69.9 | 7.7 | 19.6 | 20.6 | 209 |
| >500000 | 93.2 | 82.4 | 12.2 | 28.4 | 29.7 | 74 |
| **Income source** | | | | | | |
| Family | 89.7 | 71.7 | 8.4 | 19.3 | 20.6 | 321 |
| Scholarship | 100 | 60.0 | 10.0 | 20.0 | 50.0 | 10 |
| Tuition | 27 | 73.3 | 6.7 | 23.3 | 20.0 | 30 |
| Business | 100 | 83.3 | 6.3 | 83.3 | 50.0 | 6 |

Table S2: Knowledge of symptoms of BC among the female students who are aware of BC (N=367)

| **Variables** | **Change in the shape**  **/size in nipple** | **Lump in breast** | **Lump in axilla** | **Breast pain** | **Nipple discharge** | **Changes in the shape/size of breast** | **N** |
| --- | --- | --- | --- | --- | --- | --- | --- |
| **Age in years** | | | | | | | |
| Less than 20 | 52.2 | 52.2 | 30.4 | 52.2 | 43.5 | 39.1 | 23 |
| 20-24 | 61.7 | 56.2 | 34.5 | 61.7 | 52.4 | 45.5 | 290 |
| More than 24 | 66.0 | 50.9 | 28.3 | 66.0 | 54.7 | 39.6 | 53 |
| **Birth place** | | | | | | | |
| Rural | 59.1 | 52.2 | 32.6 | 58.3 | 49.6 | 42.6 | 230 |
| Urban | 66.4 | 59.9 | 34.3 | 67.2 | 56.9 | 47.4 | 137 |
| **Residence** | | | | | | | |
| Hall | 59.1 | 49.8 | 28.0 | 56.0 | 49.3 | 40.4 | 225 |
| Mess | 65.3 | 61.2 | 38.8 | 72.4 | 52.0 | 52.0 | 98 |
| Others | 68.2 | 68.2 | 47.7 | 65.9 | 68.2 | 47.7 | 44 |
| **Religion** | | | | | | | |
| Muslim | 62.7 | 55.3 | 32.9 | 62.1 | 53.4 | 45.3 | 322 |
| Hindus | 55.6 | 53.3 | 35.6 | 57.8 | 44.4 | 37.8 | 45 |
| **Marital status** | | | | | | | |
| Unmarried | 60.9 | 54.2 | 32.7 | 60.9 | 50.6 | 42.7 | 330 |
| married | 70.3 | 62.2 | 37.8 | 67.6 | 67.6 | 59.5 | 37 |
| **Age of menarche** | | | | | | | |
| 12-15 years | 63.7 | 55.7 | 32.5 | 62.7 | 52.2 | 44.3 | 314 |
| >15 years | 50.9 | 50.9 | 37.7 | 54.7 | 52.8 | 45.3 | 53 |
| **Family history of BC** | | | | | | | |
| Yes | 67.6 | 61.8 | 26.5 | 73.5 | 50.0 | 50.0 | 34 |
| No | 61.2 | 54.2 | 33.8 | 59.7 | 52.6 | 43.4 | 333 |
| **Family income (tk)** | | | | | | | |
| <20000 | 63.1 | 53.6 | 38.1 | 53.6 | 53.6 | 46.4 | 84 |
| 20000-50000 | 58.9 | 53.6 | 31.6 | 63.6 | 46.9 | 42.6 | 209 |
| >500000 | 68.9 | 60.8 | 32.4 | 64.9 | 51.0 | 51.4 | 74 |
| **Income source** | | | | | | | |
| Family | 61.4 | 53.6 | 30.2 | 61.4 | 51.4 | 43.9 | 321 |
| Scholarship | 90.0 | 80.0 | 60.0 | 90.0 | 60.0 | 40.0 | 10 |
| Tuition | 63.3 | 60.0 | 50.0 | 60.0 | 60.0 | 56.7 | 30 |
| Business | 33.3 | 66.7 | 66.7 | 33.3 | 50.0 | 16.7 | 6 |

Table S3: Knowledge of the risk factors of BC among the female students who are aware of BC (N=367).

| **Variables** | **M1** | **M2** | **M3** | **M4** | **M5** | **M6** | **M7** | **M8** | **M9** | **M10** | **M11** | **M12** | **N** |
| --- | --- | --- | --- | --- | --- | --- | --- | --- | --- | --- | --- | --- | --- |
| **Age in years** | | | | | | | | | | | | | |
| Less than 20 | 39.1 | 39.1 | 39.1 | 43.5 | 47.8 | 52.2 | 26.1 | 47.8 | 69.6 | 30.4 | 30.4 | 26.1 | 23 |
| 20-24 | 35.1 | 41.7 | 44.8 | 59.3 | 54.1 | 61.7 | 30.3 | 43.4 | 55.5 | 23.4 | 26.2 | 13.1 | 290 |
| More than 24 | 41.5 | 39.6 | 43.4 | 60.4 | 43.4 | 64.2 | 30.2 | 64.2 | 66.0 | 28.3 | 26.4 | 18.9 | 53 |
| **Birth place** | | | | | | |  |  |  |  |  |  |  |
| Rural | 34.8 | 42.2 | 43.5 | 59.1 | 49.1 | 61.7 | 28.7 | 50.9 | 57.8 | 23.5 | 24.8 | 13.9 | 230 |
| Urban | 40.9 | 40.1 | 46.0 | 57.7 | 57.7 | 61.3 | 32.1 | 40.1 | 58.4 | 27.0 | 29.9 | 16.1 | 137 |
| **Residence** | | | | | | |  |  |  |  |  |  |  |
| Hall | 36.4 | 36.9 | 41.8 | 53.3 | 48.9 | 56.9 | 28.4 | 44.4 | 58.2 | 20.9 | 25.8 | 12.9 | 225 |
| Mess | 38.8 | 55.1 | 51.0 | 75.5 | 63.3 | 81.6 | 35.7 | 53.1 | 53.1 | 28.6 | 29.6 | 18.4 | 98 |
| Others | 36.4 | 34.1 | 43.2 | 47.7 | 45.5 | 40.9 | 25.0 | 45.5 | 68.2 | 36.4 | 25.0 | 15.9 | 44 |
| **Religion** | | | | | | |  |  |  |  |  |  |  |
| Muslim | 37.9 | 42.5 | 44.1 | 57.1 | 51.2 | 60.9 | 30.1 | 46.6 | 58.4 | 25.5 | 25.8 | 14.3 | 322 |
| Hindus | 31.1 | 33.3 | 46.7 | 68.9 | 60.0 | 66.7 | 28.9 | 48.9 | 55.6 | 20.0 | 33.3 | 17.8 | 45 |
| **Marital status** | | | | | | |  |  |  |  |  |  |  |
| Unmarried | 37.3 | 39.4 | 43.6 | 59.4 | 52.7 | 61.5 | 29.7 | 45.2 | 55.8 | 23.9 | 26.1 | 15.2 | 330 |
| married | 35.1 | 59.5 | 51.4 | 51.4 | 48.6 | 62.2 | 32.4 | 62.2 | 78.4 | 32.4 | 32.4 | 10.8 | 37 |
| **Age of menarche** | | | | | | |  |  |  |  |  |  |  |
| 12-15 years | 37.9 | 42.7 | 45.9 | 57.0 | 54.5 | 60.5 | 28.3 | 46.2 | 58.6 | 24.2 | 26.1 | 13.7 | 314 |
| >15 years | 32.1 | 34.0 | 35.8 | 67.9 | 39.6 | 67.9 | 39.6 | 50.9 | 54.7 | 28.3 | 30.2 | 20.8 | 53 |
| **Family history of BC** | | | | | | |  |  |  |  |  |  |  |
| Yes | 29.4 | 44.1 | 35.3 | 50.0 | 47.1 | 64.7 | 29.4 | 44.1 | 58.8 | 26.5 | 29.4 | 11.8 | 34 |
| No | 37.8 | 41.1 | 45.3 | 59.5 | 52.9 | 61.3 | 30.0 | 47.1 | 58.0 | 24.6 | 26.4 | 15.0 | 333 |
| **Family income (tk)** | | | | | | |  |  |  |  |  |  |  |
| <20000 | 34.5 | 35.7 | 44.0 | 52.4 | 46.4 | 56.0 | 25.0 | 47.6 | 60.7 | 26.2 | 32.1 | 15.5 | 84 |
| 20000-50000 | 33.5 | 43.1 | 44.0 | 59.3 | 54.1 | 60.8 | 27.3 | 46.9 | 51.7 | 24.4 | 23.4 | 14.4 | 209 |
| >500000 | 50.0 | 43.2 | 45.9 | 63.5 | 54.1 | 70.3 | 43.2 | 45.9 | 73.0 | 24.3 | 29.7 | 14.9 | 74 |
| **Income source** | | | | | | |  |  |  |  |  |  |  |
| Family | 37.1 | 41.4 | 44.2 | 57.9 | 50.8 | 61.1 | 29.9 | 46.7 | 58.9 | 24.6 | 26.2 | 15.0 | 321 |
| Scholarship | 50.0 | 50.0 | 60.0 | 70.0 | 70.0 | 80.0 | 40.0 | 60.0 | 60.0 | 30.0 | 30.0 | 10.0 | 10 |
| Tuition | 30.0 | 40.0 | 43.3 | 56.7 | 56.7 | 60.0 | 30.0 | 46.7 | 46.7 | 26.7 | 30.0 | 13.3 | 30 |
| Business | 50.0 | 33.3 | 33.3 | 83.3 | 83.3 | 66.7 | 16.7 | 33.3 | 66.7 | 16.7 | 33.3 | 16.7 | 6 |

M1: Do you know aging is the risk factor for BC? M2: Do you know high fat diet is the risk factor for BC? M3: Do you know obesity is the risk factor for BC? M4: Do you know smoking is the risk factor for BC? M5: Do you know radiation to the chest is the risk factor for BC? M6: Do you know alcohol is the risk factor for BC? M7: Do you know oral contraceptive use is the risk factor for BC? M8: Do you know never breast feeding is the risk factor for BC? M9: Do you know genetic factors is the risk factor for BC? M10: Do you know never being pregnant is the risk factor for BC? M11: Do you know age at first full term pregnancy > 30years is the risk factor for BC? M12: Do you know early menarche (less 12 years) is the risk factor for BC?
